# Inhibition of PARP Sensitizes Chondrosarcoma Cell Lines to Chemo- and Radiotherapy Irrespective of the *IDH1* or *IDH2* Mutation Status

**DOI:** 10.3390/cancers11121918

**Published:** 2019-12-02

**Authors:** Sanne Venneker, Alwine B. Kruisselbrink, Inge H. Briaire-de Bruijn, Yvonne de Jong, Andre J. van Wijnen, Erik H.J. Danen, Judith V.M.G. Bovée

**Affiliations:** 1 Department of Pathology, Leiden University Medical Center, 2333 ZA Leiden, The Netherlands; 2 Department of Orthopedic Surgery, Mayo Clinic, Rochester, MN 55905, USA; 3 Division of Drug Discovery and Safety, Leiden Academic Centre for Drug Research, Leiden University, 2333 CC Leiden, The Netherlands

**Keywords:** sarcoma, chondrosarcoma, isocitrate dehydrogenase, D-2-hydroxyglutarate, AGI-5198, talazoparib, temozolomide, radiotherapy

## Abstract

Chondrosarcomas are chemo- and radiotherapy resistant and frequently harbor mutations in *isocitrate dehydrogenase* (*IDH1* or *IDH2*), causing increased levels of D-2-hydroxyglutarate (D-2-HG). DNA repair defects and synthetic lethality with poly(ADP-ribose) polymerase (PARP) inhibition occur in *IDH* mutant glioma and leukemia models. Here we evaluated DNA repair and PARP inhibition, alone or combined with chemo- or radiotherapy, in chondrosarcoma cell lines with or without endogenous *IDH* mutations. Chondrosarcoma cell lines treated with the PARP inhibitor talazoparib were examined for dose–response relationships, as well as underlying cell death mechanisms and DNA repair functionality. Talazoparib was combined with chemo- or radiotherapy to evaluate potential synergy. Cell lines treated long term with an inhibitor normalizing D-2-HG levels were investigated for synthetic lethality with talazoparib. We report that talazoparib sensitivity was variable and irrespective of *IDH* mutation status. All cell lines expressed Ataxia Telangiectasia Mutated (ATM), but a subset was impaired in poly(ADP-ribosyl)ation (PARylation) capacity, homologous recombination, and *O-6-methylguanine-DNA methyltransferase* (*MGMT*) expression. Talazoparib synergized with temozolomide or radiation, independent of IDH1 mutant inhibition. This study suggests that talazoparib combined with temozolomide or radiation are promising therapeutic strategies for chondrosarcoma, irrespective of *IDH* mutation status. A subset of chondrosarcomas may be deficient in nonclassical DNA repair pathways, suggesting that PARP inhibitor sensitivity is multifactorial in chondrosarcoma.

## 1. Introduction

Chondrosarcomas are malignant cartilage producing tumors and are the third most common bone malignancy [1]. These tumors can be classified into different subtypes: Conventional chondrosarcoma (85%), dedifferentiated chondrosarcoma (10%), and rare subtypes (5%), including mesenchymal-, clear cell-, and periosteal chondrosarcoma. Conventional chondrosarcoma can be further subdivided into central (in the medulla of the bone, 85%) and peripheral (on the bone surface, 15%) tumors. Histological grading is the most important prognostic factor for predicting survival of conventional chondrosarcoma patients. Patients with atypical cartilaginous tumors (ACT)/chondrosarcoma grade I have an overall 10-year survival rate of 88%, while patients with a grade II or grade III chondrosarcoma have a reduced survival rate of 62% and 26%, respectively [2]. These survival rates are partly limited by available treatment options, because chondrosarcomas are intrinsically resistant towards conventional chemo- and radiotherapy and targeted therapies are not yet available. At present, surgery remains the only available curative treatment option [3]. These therapeutic limitations emphasize the importance of the development of novel therapeutic strategies, especially for unresectable conventional chondrosarcoma.

Central conventional chondrosarcomas harbor specific point mutations in *isocitrate dehydrogenase 1* or *-2* (*IDH1* or *IDH2*) in ~50% of the cases [4,5]. These enzymes function in the tricarboxylic acid cycle (TCA cycle) in which they convert isocitrate into α-ketoglutarate (α-KG) and CO_2_. The specific point mutations in the arginine residues (R132 for *IDH1* and R140/172 for *IDH2*) are gain-of-function mutations and induce the additional conversion of α-KG into the oncometabolite D-2-hydroxyglutarate (D-2-HG), leading to elevated levels of this oncometabolite [6]. The most frequently observed point mutation in chondrosarcoma is the *IDH1*^R132C^ (~40%), which produces relatively high levels of D-2-HG [7]. Other point mutations that are commonly found in chondrosarcoma are *IDH1*^R132G^, *IDH1*^R132H^*, IDH2^R172S^, IDH1*^R132L^, and *IDH1^R132S^* [4]. The structural similarity between α-KG and D-2-HG is high and therefore the oncometabolite competitively inhibits α-KG dependent enzymes, such as DNA- and histone demethylases, leading to epigenetic alterations like DNA hypermethylation [8]. Furthermore, mutations in *IDH1* or *IDH2* (collectively referred to as *IDH*) cause alterations in cell metabolism, cell growth signalling pathways, and the DNA damage response [9,10]. However, we have shown that direct inhibition of the IDH1 mutant enzyme with AGI-5198 does not change the tumorigenic properties of chondrosarcoma cell lines *in vitro*, suggesting that these tumors become independent of their *IDH* mutation over time [11]. As an alternative, the underlying alterations induced by *IDH* mutations might provide a vulnerability that could be therapeutically exploited. 

Several studies have examined synthetic lethal interactions with *IDH* mutations. Synthetic lethality is based on the principle that alterations in two genes induce a lethal phenotype, while individual alteration of these genes has no effect on cell viability. Most of these studies were performed in acute myeloid leukaemia (AML) and glioma, both of which also harbor *IDH* mutations [12,13]. Several compounds have synthetic lethal phenotypes with *IDH* mutations, including agents that induce DNA damage or target B-cell lymphoma 2 (Bcl-2) family members, nicotinamide phosphoribosyltransferase (NAMPT), glutaminase, poly(ADP-ribose) polymerase (PARP) and DNA (cytosine-5)-methyltransferase 1 (DNMT1) [14,15,16,17,18,19,20,21,22,23]. One of these targets is PARP, a protein involved in the detection and repair of single-strand DNA breaks. Potential mechanisms underlying this synthetic lethal interaction are a reduced expression of Ataxia Telangiectasia Mutated (ATM), as well as D-2-HG dependent inhibition of lysine-specific demethylase 4A and 4B (KDM4A and KDM4B) and the homologous recombination pathway [15,20,21]. Therefore, this study evaluated PARP inhibition and the functionality of DNA repair pathways in endogenous *IDH* mutant and *IDH* wildtype chondrosarcoma cell lines. Furthermore, we explored if PARP mediates resistance to chemo- and radiotherapy in chondrosarcoma. Our experimental design focused on talazoparib, because it is one of the most potent, FDA-approved PARP inhibitors that causes both catalytic inhibition and DNA trapping of PARP (i.e., ~100 fold more than olaparib) [24]. This dual role increases the level of induced DNA damage and may overcome the intrinsic chemo- and radiotherapy resistance in chondrosarcoma.

## 2. Results

### 2.1. Chondrosarcoma Cell Lines Are Variably Sensitive to PARP Inhibition, Irrespective of the IDH Mutation Status

**Figure 1:**
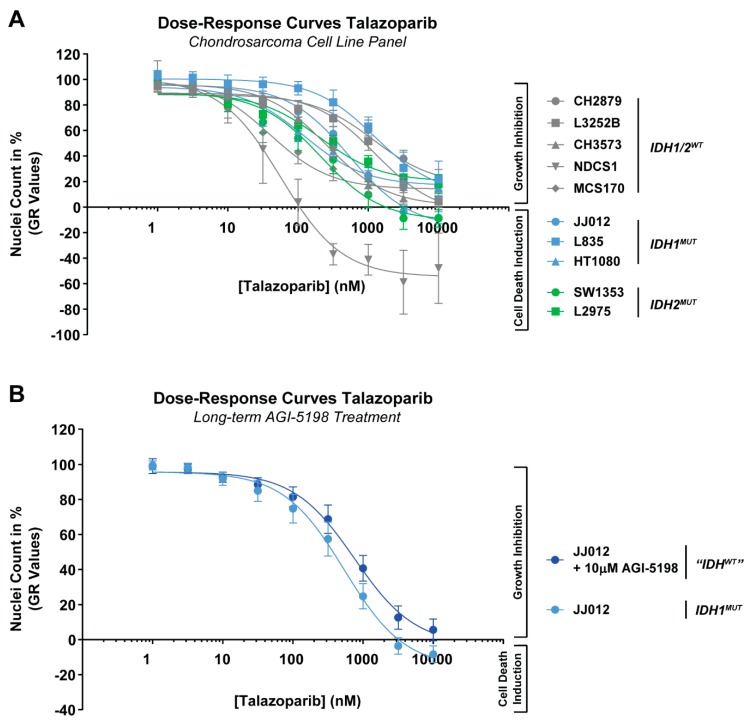
Chondrosarcoma cell lines are variably sensitive to poly(ADP-ribose) polymerase (PARP) inhibition, irrespective of the *isocitrate dehydrogenase* (*IDH*) mutation status. (**A**) Dose-response curves of talazoparib after 72 h of treatment for 10 chondrosarcoma cell lines. Talazoparib sensitivity differs among cell lines. (**B**) Long-term AGI-5198 treatment (>20 passages) could not significantly rescue the effect of talazoparib treatment in an *IDH1* mutant cell line. A Kruskal–Wallis/Dunn’s test was performed to determine significant changes in nuclei count between matching talazoparib concentrations. Dose-response curves were corrected for growth rate and GR_50_ values were calculated. Data points represent the mean of three experiments performed in triplicate ± standard deviation.

To assess PARP inhibitor sensitivity, we generated dose-response curves with talazoparib for 10 chondrosarcoma cell lines. Chondrosarcoma cell lines were variably sensitive to PARP inhibition with growth rate corrected IC_50_ (GR_50_) values ranging from 34 nM to >1000 nM after 72 h of treatment (Figure 1A and Table 1). A subset of chondrosarcoma cell lines (NDCS1, MCS170, SW1353, and HT1080) showed a similar sensitivity to PARP inhibition as described in literature for cell lines with impaired DNA repair pathways (i.e., IC_50_ values between 0.1 and 100 nM) (Table 1) [25,26,27]. Talazoparib inhibited the growth of the cells present before the start of the 72-h drug treatment (i.e., time 0 measurement is set at 0%) in most chondrosarcoma cell lines (Figure 1A), although cell death in this pre-existing cell population can be induced in almost all chondrosarcoma cell lines at infinite drug concentrations (GR_Inf_ values) (Table 1). Sensitivity to talazoparib was not correlated to *IDH* mutation status (Figure 1A) and long-term treatment with the IDH1 mutant inhibitor AGI-5198 did not significantly rescue the effect of talazoparib in the *IDH1* mutant (*IDH1^MUT^)* cell line JJ012 (Figure 1B). Thus, chondrosarcoma cells exhibited differences in sensitivity to PARP inhibition, regardless of the *IDH* mutation status.

### 2.2. PARP Inhibition Minimally Induces Apoptosis and Causes a G2/M Phase Cell Cycle Arrest in Chondrosarcoma Cell Lines

**Figure 2:**
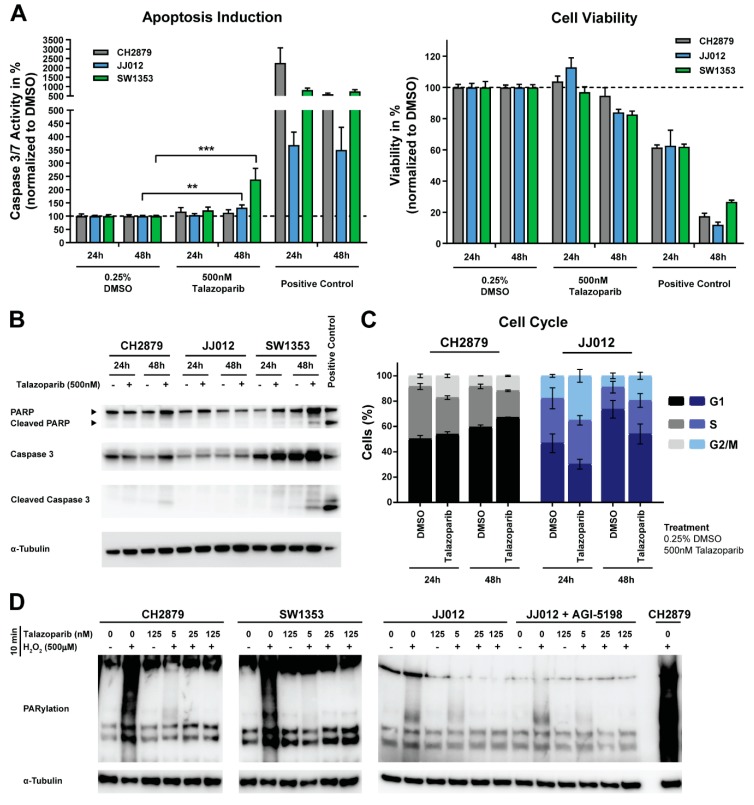
PARP inhibition minimally induced apoptosis, caused a Gap 2/Mitosis (G2/M) cell cycle arrest, and blocked poly(ADP-ribosyl)ation (PARylation) in chondrosarcoma cell lines. (**A**) Caspase 3/7 activity and corresponding viability after 24 h and 48 h treatment with 500 nM talazoparib. A 5 µM ABT-737 + 1 µM doxorubicin was used as positive control. SW1353 and JJ012 cells showed a significant induction of apoptosis after 48 h talazoparib treatment. Bars represent the mean of three experiments performed in duplicate ± standard deviation. Significant changes towards DMSO controls were determined with a Kruskal–Wallis/Dunn’s test at * *p* < 0.05, ** *p* < 0.01, *** *p* < 0.001, **** *p* < 0.0001. (**B**) Western blot for cleaved PARP and cleaved caspase 3 after 24 h and 48 h treatment with talazoparib. Apoptosis was observed in CH2879 and SW1353 cells after 48 h treatment. CH2879 cells treated for 24 h with 5 µM ABT-737 and 1 µM doxorubicin was used as positive control. The α-tubulin was used as a loading control. Whole blot with densitometry readings can be found in Figure A2A. (**C**) Cell cycle analysis after 24 h and 48 h treatment with 500 nM talazoparib. Both chondrosarcoma cell lines showed an increase in the G2/M phase. Bars represent the mean of three independent experiments ± standard deviation. (**D**) Western blot for PARylation after DNA damage induction by exposure to 500 µM H_2_O_2_ −/+ talazoparib for 10 min. The 5 nM talazoparib treatment blocked PARylation in all cell lines. JJ012 showed a reduced PARylation capacity. The α-tubulin was used as a loading control. Whole blots with densitometry readings can be found in Figure A2B.

Three central conventional chondrosarcoma cell lines with an *IDH* wildtype (CH2879) or an endogenous *IDH1^R132G^* mutation (JJ012) or *IDH2^R172S^* mutation (SW1353) were selected to elucidate the underlying growth inhibition or cell death mechanism. Cell lines were treated with 500 nM talazoparib, which reflects the GR_80_ value of SW1353 after 72 h of treatment to enable cell line comparisons and to avoid toxic side effects. Caspase 3/7 activity was significantly induced in JJ012 and SW1353 cells after 48 h treatment with 500 nM talazoparib, although the observed effect was minimal as compared to the positive control (Figure 2A). Western blotting for cleaved PARP and cleaved caspase 3 confirmed this induction of apoptosis in SW1353 cells and showed a minimal induction of cleaved caspase 3 in CH2879 cells (Figure 2B). Furthermore, cell cycle analysis showed that treatment with 500 nM talazoparib for 24 h or 48 h arrested CH2879 and JJ012 cells in the Gap 2/Mitosis (G2/M) cell cycle phase, with a concomitant reduction in the fraction of cells in S-phase (CH2879) or G1 phase (JJ012) (Figure 2C). This finding indicates that talazoparib affected chondrosarcoma growth by induction of apoptosis or a cell cycle arrest. 

### 2.3. JJ012 Cells Have a Reduced Capacity to Sense DNA Damage, Independent of the IDH1 Mutation

To confirm the on-target PARP inhibitory effect of talazoparib, poly(ADP-ribosyl)ation (PARylation) was assessed after H_2_O_2_ treatment. Short-term treatment (i.e., 10 minutes) with nanomolar concentrations of talazoparib blocked the formation of PAR chains in all cell lines, indicating that PARP function was almost completely inhibited at 5 nM talazoparib (Figure 2D). However, the PARylation capacity of JJ012 cells was reduced, suggesting an underlying defect in sensing DNA damage. The diminished PARylation capacity of JJ012 cells could not be reversed by long-term IDH1 mutant inhibition (Figure 2D). Hence, JJ012 chondrosarcoma cells may have a deficiency in sensing DNA damage, although irrespective of the *IDH1* mutation.

### 2.4. Chondrosarcoma Cell Lines Are Homologous Recombination Proficient and Maintain Nominal Expression of ATM

**Figure 3:**
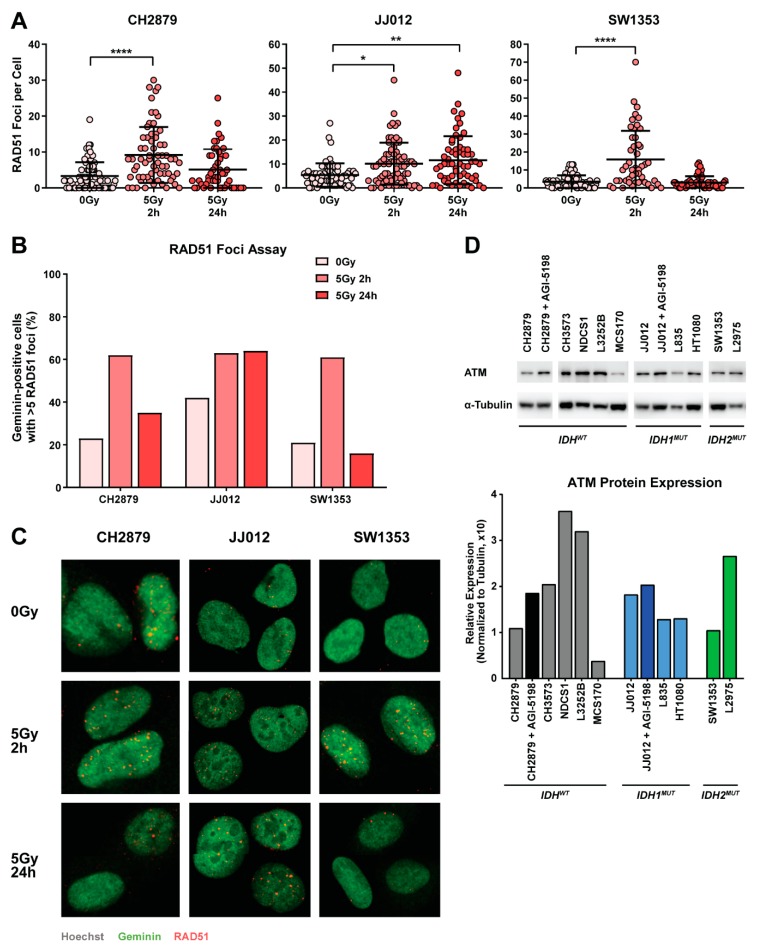
Chondrosarcoma cell lines are homologous recombination proficient and maintain nominal expression of Ataxia Telangiectasia Mutated (ATM). (**A**) The amount of RAD51 foci per geminin-positive cell after 2 h or 24 h recovery of 5 Gy γ-radiation treatment. All cell lines showed an induction of RAD51 foci 2 h after treatment. JJ012 cells retained DNA damage signalling after 24 h. Quantification was performed with a previously published ImageJ macro [29]. Every data point represents a geminin-positive cell. The mean ± standard deviation is depicted per treatment group. Significant changes towards 0 Gy controls were determined with a Kruskal–Wallis/Dunn’s test at * *p* < 0.05, ** *p* < 0.01, *** *p* < 0.001, **** *p* < 0.0001. (**B**) The percentage of geminin-positive cells with >5 RAD51 foci. Cut-offs for homologous recombination status after 2 h recovery of 5 Gy γ-radiation treatment: 0–20% is impaired, 20–50% is intermediate, and 50–100% is normal. All cell lines showed activation of the homologous recombination pathway. JJ012 cells retained activation of the DNA repair pathway after 24 h. (**C**) Representative images per treatment condition of several single cells. (**D**) Western blot for ATM expression. ATM expression was not correlated with *IDH* mutation status. The α-tubulin was used as a loading control. All samples were loaded on the same gel. Whole blot with densitometry readings can be found in Figure A2C. Graph represents the quantification of ATM expression normalized to α-tubulin.

To determine if chondrosarcoma cell lines are deficient in homologous recombination, a RAD51 foci assay was performed [28]. After a 2 h recovery of γ-radiation treatment, all cell lines showed a significant induction of RAD51 foci, indicative of a proficient homologous recombination pathway (Figure 3A). However, 24 h after irradiation, CH2879 and SW1353 cells each exhibited evidence of recovery as reflected by partial repair of DNA damage, while JJ012 cells retained DNA damage signals (Figure 3A). This difference in DNA repair was confirmed by the amount of geminin positive cells with >5 RAD51 foci, because only JJ012 retained a high percentage of RAD51 positive cells over time (Figure 3B,C). It was previously shown by other investigators that expression of the DNA repair initiator ATM is reduced in *IDH* mutant cells [15,21]. However, no difference in ATM expression was observed between the *IDH^WT^* and *IDH^MUT^* chondrosarcoma cell lines used in our study. Moreover, ATM expression was not enhanced in JJ012 cells after long-term AGI-5198 treatment (Figure 3D). Hence, differences in DNA repair that were independent of ATM expression were evident among the three lines that we examined.

### 2.5. The Combination of PARP Inhibition and Temozolomide Is Synergistic in Chondrosarcoma Cell Lines

**Figure 4:**
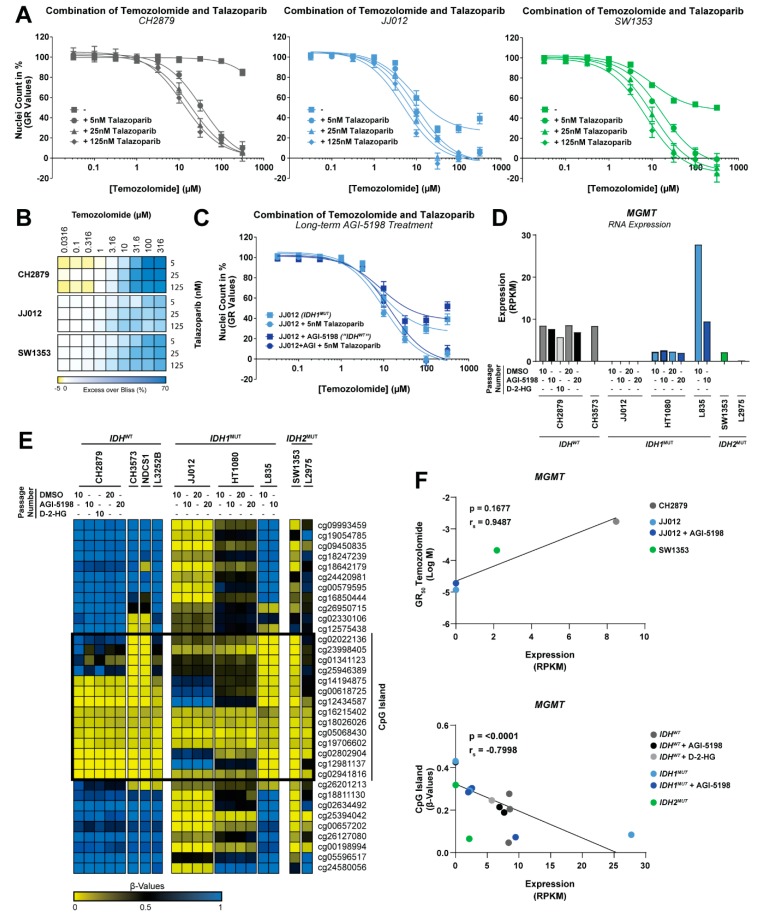
Combination of PARP inhibition and temozolomide is synergistic in chondrosarcoma cell lines. (**A**) Dose-response curves for single temozolomide treatment or temozolomide combined with talazoparib. Talazoparib sensitized all cell lines towards temozolomide. Dose-response curves were corrected for growth rate. Data points represent the mean of three experiments performed in triplicate ± standard deviation. (**B**) Heatmap represents the calculated Excess over Bliss scores for temozolomide and talazoparib combinations, in which yellow represents antagonism, white represents additivity, and blue represents synergy. Combination induced synergy in all tested cell lines. (**C**) Long-term AGI-5198 treatment (>20 passages) could not rescue the effect of single or combined treatments in an *IDH1* mutant cell line. (**D**) RNA expression of *MGMT* per cell line determined from a previously published RNA sequencing data set [11]. Long-term AGI-5198 (1.5 µM) or D-2-HG (5 mM) treatment did not influence *MGMT* RNA expression. (**E**) *MGMT* promoter methylation determined from a previously reported genome-wide methylation data set [11]. Heatmap represents the β-values, in which yellow represents low methylation and blue represents high methylation. (**F**) Spearman correlations between temozolomide sensitivity, *MGMT* expression, and *MGMT* promoter methylation status. A trend towards a correlation between *MGMT* expression and temozolomide sensitivity was observed. Promoter methylation significantly correlates to *MGMT* RNA expression.

**Figure A1:**
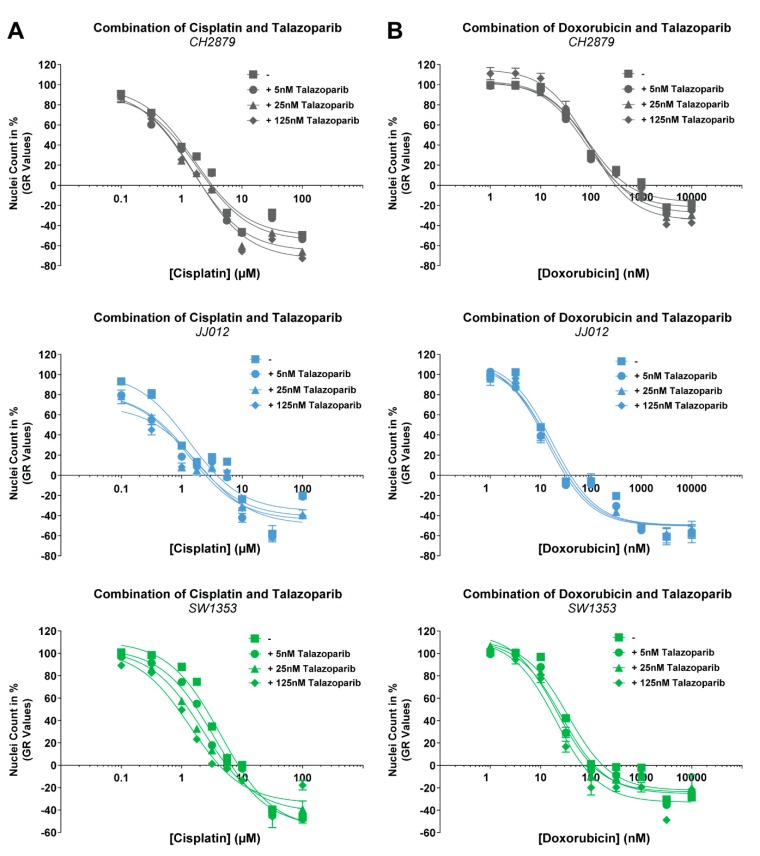
Combination of talazoparib and cisplatin or doxorubicin does not induce a synergistic effect in chondrosarcoma cell lines. (**A**) Dose-response curves for single cisplatin treatment or cisplatin combined with talazoparib. None of the cell lines showed sensitization towards cisplatin. (**B**) Dose-response curves for single doxorubicin treatment or doxorubicin combined with talazoparib. Talazoparib did not sensitize cells to doxorubicin treatment. Dose-response curve data was corrected for growth rate. Data points represent the mean of one experiment performed in triplicate ± standard deviation.

We next examined if PARP inhibition could overcome the intrinsic chemo- and radiotherapy resistance present in chondrosarcoma. Talazoparib was combined with increasing concentrations of two conventional chemotherapeutic agents (cisplatin and doxorubicin), and temozolomide, a DNA alkylating/methylating agent known for its synergistic effect with PARP inhibitors [30,31]. No synergy was observed when talazoparib was combined with cisplatin or doxorubicin (Figure A1, Appendix A). However, combination of talazoparib with temozolomide showed synergy in all three chondrosarcoma cell lines analyzed as determined by the Excess over Bliss score (Figure 4A,B). Notably, while JJ012 and SW1353 cells responded to single temozolomide treatment, CH2879 cells were resistant to >100 uM temozolomide but became sensitive to <10 uM temozolomide in the presence of talazoparib. Long-term AGI-5198 treatment of JJ012 cells did not alter the sensitivity towards single or combination therapies with temozolomide (Figure 4C). This result indicates that the effect of both therapeutic strategies was independent of *IDH* mutation status. 

Temozolomide sensitivity in glioblastoma patients correlates with the expression and promoter methylation status of O-6-methylguanine-DNA methyltransferase (*MGMT*) [32], an enzyme that counteracts temozolomide-induced DNA damage. *IDH* mutations cause hypermethylation of DNA in tumors, including chondrosarcoma [5], and this epigenetic modification may reduce *MGMT* mRNA expression [33]. Therefore, we investigated the methylation status of the *MGMT* promoter and effects on RNA expression in our chondrosarcoma cell line panel with or without long-term treatment with AGI-5198 or D-2-HG. Expression of *MGMT* was higher in CH2879 cells as compared to JJ012 and SW1353 cells and Spearman correlation analysis showed a trend towards an association between expression and temozolomide GR_50_ values in these cell lines, although sample size was small (Figure 4D,F). Furthermore, a significant correlation between *MGMT* expression and CpG island methylation status was observed (Figure 4E,F). However, long-term treatment with AGI-5198 in *IDH1* mutant cell lines or with the oncometabolite D-2-HG in a wildtype cell line did not affect *MGMT* RNA expression (except for L835) or promoter methylation (Figure 4D,E). Overall, these data indicated that the effectivity of temozolomide as either single or combination therapy in chondrosarcoma is not determined by the *IDH* mutation status and may be associated with other molecular differences between cell lines, such as *MGMT* expression.

### 2.6. PARP Inhibition Sensitizes Chondrosarcoma Cells to Radiation Which is Partially Rescued when Mutant IDH1 is Inhibited

**Figure 5:**
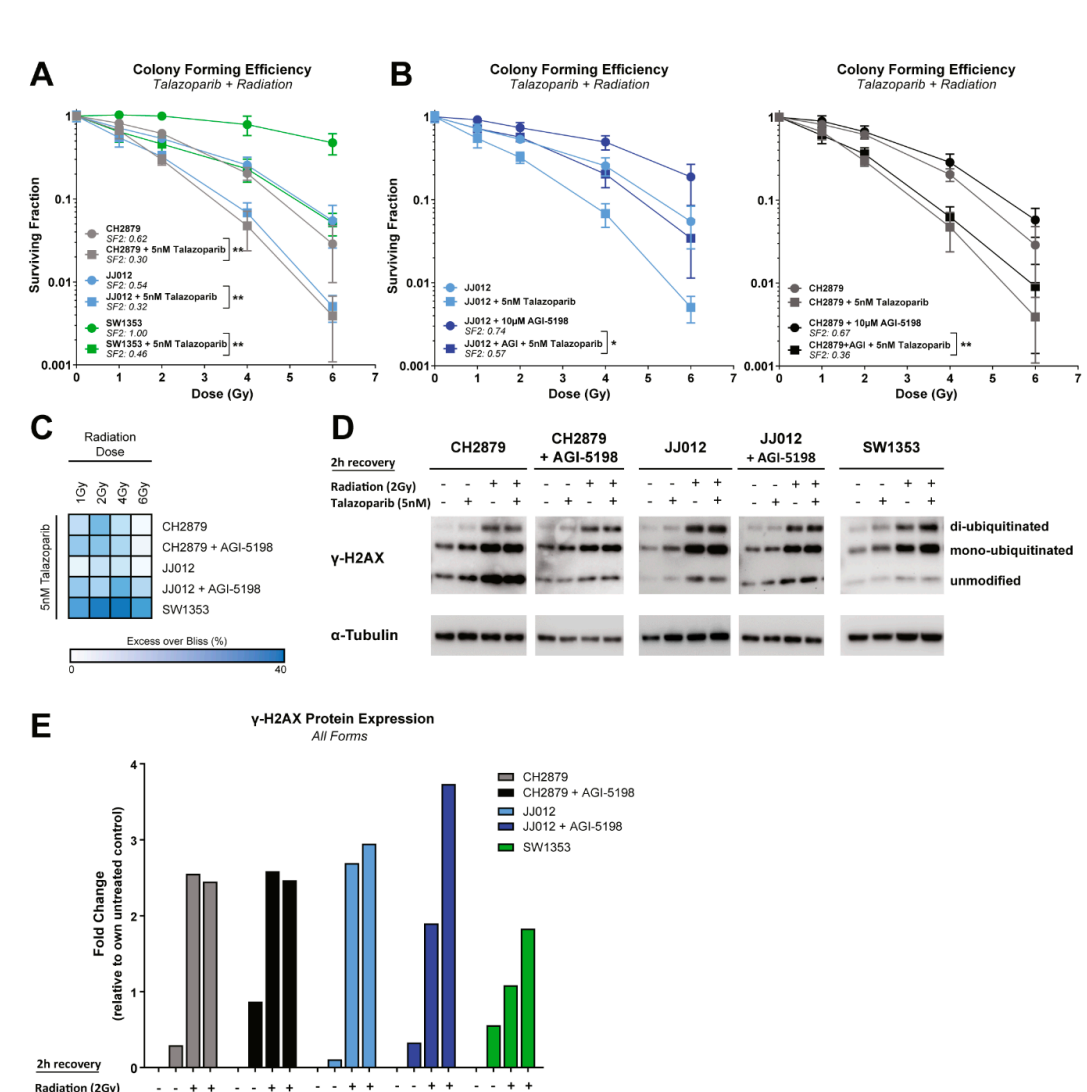
PARP inhibition sensitized chondrosarcoma cells to radiation which was partially rescued when mutant IDH1 was inhibited. (**A**) Surviving fractions of three chondrosarcoma cell lines after treatment with single dosages of γ-radiation ± 5 nM talazoparib. Talazoparib sensitized all cell lines towards radiotherapy. (**B**) Surviving fractions of long-term AGI-5198 treated cell lines after treatment with single dosages of γ-radiation ± 5 nM talazoparib. A partial rescue was observed in JJ012 + AGI-5198 cells, while CH2879 + AGI-5198 cells gave a similar response as the nontreated counterpart. Data points represent the mean of two experiments performed in triplicate ± standard deviation. Significant changes between SF2 values of ± 5 nM talazoparib treated counterparts were determined with a Mann–Whitney test at * *p* < 0.05, ** *p* < 0.01, *** *p* < 0.001, **** *p* < 0.0001. (**C**) Heatmap represents the calculated Excess over Bliss scores for radiotherapy and talazoparib combinations, in which white represents additivity and blue represents synergy. Combination induced synergy in all tested cell lines. (**D**) Western blot for γ-H2AX expression after 2 h recovery of 2 Gy γ-radiation treatment. All cell lines showed induction of DNA damage signaling. The α-tubulin was used as a loading control. Whole blots with densitometry readings can be found in Figure A2D. (**E**) Quantification of the expression of all γ-H2AX forms. Per sample, γ-H2AX expression was normalized to α-tubulin expression. For each cell line, fold changes were calculated relative to the untreated control.

To determine if there is a synergistic effect between talazoparib and γ-irradiation, colony formation assays were performed. Combinations of talazoparib and radiotherapy resulted in a significant reduction of the surviving fraction after 2 Gy (SF2) in all cell lines, indicating that this combination therapy could overcome radiotherapy resistance in chondrosarcoma (Figure 5A) [34].

Interestingly, long-term AGI-5198 treatment in JJ012 cells induced a partial rescue of the effect after both single and combined treatment strategies. This radiotherapy desensitizing effect was not observed when CH2879 cells were treated long-term with AGI-5198, suggesting a specific on-target IDH1 mutant inhibition effect (Figure 5B). In all cell lines, combination of γ-irradiation and talazoparib led to synergistic effects indicated by the Excess over Bliss score (Figure 5C). Furthermore, radiation alone or radiation combined with talazoparib induced DNA damage in all cell lines as indicated by induction of γ-H2AX signalling at 2 h after treatment. Of note, combination therapy only induced additional DNA damage in the two most radio-resistant cell lines (i.e., SW1353 and JJ012 + AGI-5198) (Figure 5D,E).

## 3. Discussion

In this study, we explored if PARP inhibition alone or in combination with chemo- or radiotherapy could be a potential therapeutic strategy for chondrosarcoma and whether sensitivity towards these therapies depends on the *IDH* mutation status. Furthermore, we assessed if chondrosarcomas have impaired DNA repair pathways.

A synthetic lethal interaction between *IDH* mutations and PARP inhibition was identified in glioma and AML, and ascribed to decreased ATM expression in AML [15,20,21]. We did not identify a similar association in a panel of human chondrosarcoma cell lines. Likewise, the synthetic lethal interaction between temozolomide and *IDH* mutations described in gliomas was not observed in our study [35,36]. Our research group published that the reported synthetic lethal interaction between *IDH* mutations and Bcl-2, NAMPT, and glutaminase inhibition was absent in chondrosarcoma [11,37,38,39]. Together, these findings indicate that therapeutic strategies in AML and glioma based on *IDH* mutation status cannot be directly translated to chondrosarcoma. 

Several considerations may clarify differences in results among the above studies that examined synthetic lethal interactions with *IDH* mutations. First, distinct tumor types differ in endogenous gene expression patterns, mutational background, and tumor microenvironment (i.e., hypoxia in chondrosarcoma), which probably influences the role of *IDH* mutations in tumor onset and progression. Our current and previous [11] findings suggest that *IDH* mutations may be important in the onset but become less relevant in advanced chondrosarcomas. Second, the most frequent *IDH* mutations in glioma and AML (*IDH1^R132H^* and *IDH2^R140Q^*, respectively) are weak D-2-HG producers as compared to the most common point mutation in chondrosarcoma (*IDH1^R132C^)* [7]. The biological effects of the *IDH* mutation may depend on the level of D-2-HG, which could explain why not all *IDH*-mutated tumors have identical targetable vulnerabilities. Third, most synthetic lethal interactions were identified in *in vitro* models with introduced *IDH* mutations. These models do not fully represent the tumor type or the genetic background in which an *IDH* mutation normally exists. Finally, the genetic background in which the *IDH* mutation functions determines whether gliomas will be resistant or sensitive to radiotherapy, because concomitant loss of *TP53* and *alpha thalassemia/mental retardation syndrome X-linked gene (ATRX)* results in a radio-resistant phenotype of *IDH*-mutated gliomas [40]. These considerations emphasize the importance of conducting studies for the identification of synthetic lethal interactions in *in vitro* models with endogenous *IDH* mutations. 

In this study, we found that chondrosarcoma cell lines are variably sensitive towards single PARP inhibitor treatment irrespective of *IDH* mutation status, with 4 out of 10 cell lines harboring similar GR_50_ or IC_50_ values as cell lines with known DNA repair deficiencies [25,26,27]. The chondrosarcoma cell lines had very different growth rates, which rendered the interpretation of drug sensitivity assays inaccurate unless growth rate corrections were applied. As shown in a previous study [41] and the current study (Table 1), correction for growth rates may change the estimated IC_50_, and this growth rate dependence necessitates numerical corrections in drug effects to improve comparisons between *in vitro* studies, *in vitro* to *in vivo* translations and the identification of molecular mechanisms that mediate vulnerabilities to different therapeutic strategies. 

A RAD51 foci assay shows that CH2879 and SW1353 cells have a functioning homologous recombination pathway, indicating that PARP inhibitor sensitivity in chondrosarcoma partly depends on a different mechanism than classical homologous recombination pathway defects, such as *BRCA* mutations. Indeed, such alternative scenarios have been described: For instance, PARP signalling attracts chromatin remodelers and is linked to changes in several DNA and histone epigenetic modifications (e.g., acetylation and methylation) and thus controls the epigenetic landscape of cells [42,43]. Of note, JJ012 cells lack efficiency to repair DNA double-strand breaks that are localized in RAD51 foci within 24 h, indicative of a delay in DNA repair or an impairment in the disassembly of RAD51 and other repair factors from DNA double-strand breaks [44]. These findings correlate with γ-H2AX foci staining previously published by our group in which we showed that only JJ012 cells retained DNA damage signalling over time [45]. Additionally, JJ012 cells have a lower capacity to sense DNA damage as indicated by the reduced formation of PAR chains which recruit DNA repair factors [46]. Moreover, long-term AGI-5198 treatment in JJ012 cells led to a partial rescue of the radiotherapy effect resulting in a more resistant phenotype. These findings suggest that in a subset of chondrosarcomas PARP inhibitor sensitivity can be explained by a nonclassical DNA damage repair deficiency, such as low capacity to sense DNA damage or defects more downstream in the homologous recombination pathway.

We also explored the role of PARP in the intrinsic chemo- and radiotherapy resistance observed in chondrosarcoma. Our results show that combination therapies with temozolomide or radiotherapy and PARP inhibition were synergistic in all chondrosarcoma cell lines and are therefore promising candidate therapeutic strategies for both *IDH^WT^* and *IDH^MUT^* chondrosarcoma patients. Recently, a similar radio-sensitizing effect was described in a study in which olaparib was combined with different radiotherapeutic strategies in the chondrosarcoma cell line CH2879 [47]. Notably, combination of talazoparib with the DNA alkylating/methylating agent temozolomide seems to be most effective. The mechanism underlying the observed synergy is probably related to PARP trapping on DNA; a known mechanism of temozolomide to enhance PARP inhibitor effectiveness [30,48]. Furthermore, temozolomide sensitivity in chondrosarcoma cell lines appears to be associated with *MGMT* RNA expression and methylation of the CpG island located in the promoter. It is conceivable that *MGMT* expression or promoter methylation could be viable biomarkers for temozolomide mono- or combination therapy in chondrosarcoma. 

Although talazoparib and temozolomide are currently FDA approved, both are used for different indications and are usually given as a monotherapy. Talazoparib is approved for the treatment of patients with germline BRCA-mutated HER2-negative locally advanced or metastatic breast cancer, reaching plasma concentrations of 55 nM after multiple daily dosing of 1 mg [49]. Temozolomide is currently approved for newly diagnosed glioblastoma multiforme and refractory anaplastic astrocytoma patients, reaching plasma concentrations of 30 µM after multiple daily dosing of 150 mg/m^2^ [50]. The *in vitro* experiments in this study were performed with drug concentrations in the range of the observed clinical maximum plasma concentrations, suggesting that the observed effects are specific and not related to off-target toxicities. However, *in vitro* models do not represent tumor heterogeneity, the high complexity of the tumor microenvironment and the interplay between different cell types, underlining the need for thorough *in vivo* testing before findings can be translated into the clinic. The present *in vitro* results are promising and give a rationale for extensive testing in 3D *in vitro* models and orthotopic *in vivo* models of chondrosarcoma.

## 4. Materials and Methods 

### 4.1. Compounds

The PARP inhibitor talazoparib (S7048, Selleckchem, Houston, TX, USA), the IDH1 mutant (R132H and R132C) inhibitor AGI-5198 (14624, Cayman Chemical, Ann Arbor, MI, USA), the alkylating agent temozolomide (S1237, Selleckchem) and the Bcl-xL/Bcl-2/Bcl-w inhibitor ABT-737 (S1002, Selleckchem) were dissolved in DMSO. The cell permeable derivative of D-2-HG, (2R)-Octyl-α-hydroxyglutarate (16366, Cayman Chemical), was dissolved in phosphate buffered saline (PBS). Doxorubicin (2 mg/mL in 0.9% NaCl) and cisplatin (1 mg/mL in 0.9% NaCl) were obtained from Leiden University Medical Center (in-house hospital pharmacy) and diluted in PBS. Hydrogen peroxide (H_2_O_2_) (Merck, Darmstadt, Germany) was also diluted in PBS.

### 4.2. Cell Culture

The central conventional chondrosarcoma cell lines CH2879 (*IDH* wildtype (*IDH*^WT^)) [5,51], JJ012 (*IDH1*^R132G^) [5,52], and SW1353 (ATCC, *IDH2*^R172S^) [5] were cultured in Roswell Park Memorial Institute (RPMI) 1640 medium (Gibco, Invitrogen Life-Technologies, Scotland, UK) supplemented with 10% heat-inactivated Fetal Bovine Serum (FBS) (F7524, Sigma-Aldrich, Saint Louis, MO, USA). The central conventional chondrosarcoma cell lines CH3573 (*IDH*^WT^) [53] and L835 (*IDH1*^R132C^) [5,54] were cultured in RPMI 1640 + 20% FBS. The dedifferentiated chondrosarcoma cell lines NDCS1 (*IDH*^WT^) [5,55], HT1080 (*IDH1*^R132C^) [11,56], and L2975 (*IDH2*^R172W^) [5,54] were cultured in RPMI 1640 + 10% FBS and L3252B (*IDH*^WT^) [54] was cultured in RPMI 1640 + 20% FBS. The mesenchymal chondrosarcoma cell line MSC170 (*IDH*^WT^) [57] was cultured in Iscove’s Modified Dulbecco’s Medium (IMDM) (Gibco, Invitrogen Life-Technologies) supplemented with 15% FBS. Long-term AGI-5198 treated cell lines (20 passages with 1.5 µM AGI-5198) [11] were cultured in normal growth medium + 10 µM AGI-5198. All cell lines were cultured in a humidified incubator with 5% CO_2_ at 37 °C. Short Tandem Repeat (STR) profiling (GenePrint 10 System, Promega, Madison, WI, USA) and PCR-based mycoplasma tests were performed monthly.

### 4.3. Cell Viability and Nuclei Count Assays

Chondrosarcoma cell lines were seeded in 96 well plates (3 × 10^3^ to 15 × 10^3^ cells/well) and were allowed to attach overnight. Cells were treated with different compounds (i.e., talazoparib, temozolomide, cisplatin, and doxorubicin) in concentrations ranging from 0 to 316 µM. The solvents DMSO and PBS were used as negative controls. After 72 h of treatment, cell viability was measured with PrestoBlue cell viability reagent (A13262, Invitrogen Life-Technologies) according to the manufacturer’s protocol. Fluorescence was measured at 560/590 nm with the Victor3V 1420 multilabel counter (Perkin Elmer, Groningen, The Netherlands) after 1 to 1.5 h of incubation at 37 °C. Subsequently, cells were fixed with 4% formaldehyde (Q Path, VWR Chemicals, Radnor, PA, USA) and stained with 2 µg/mL Hoechst 33342 (H1399, Invitrogen Life-Technologies). Nuclei were counted with the Cellomics ArrayScan VTI HCS 700 series and HCS Studio Cell Analysis Software (ThermoFisher Scientific, Waltham, MA, USA). To correct for growth rate, data were normalized to the “time 0 measurement” (i.e., cell viability or nuclei count before treatment) with GR Calculator (http://www.grcalculator.org) [41]. Experiments were performed in triplicate and repeated three times.

### 4.4. Apoptosis Assay

CH2879, JJ012, and SW1353 cells were seeded in white 96 well plates (3 × 10^3^ to 7 × 10^3^ cells/well). After overnight attachment, cultures were treated with 500 nM talazoparib for 24 h or 48 h. The solvent DMSO was used as a negative control and a combination of 5 µM ABT-737 and 1 µM doxorubicin was used as a positive control. After 24 h or 48 h of treatment, the CaspaseGlo 3/7 Assay (Promega) was used according to the manufacturer’s protocol to measure apoptosis induction. Luminescence was measured with a Victor3V 1420 multilabel counter (Perkin Elmer) after 30 min of incubation at room temperature. Experiments were performed in duplicate and repeated three times.

### 4.5. Cell Cycle Assay

CH2879, JJ012, and SW1353 cells were seeded in 6 well plates (9 × 10^4^ to 21 × 10^4^ cells/well). After overnight attachment, cultures were treated with 500 nM talazoparib or the solvent control DMSO for 24 h or 48 h. After the indicated treatment times, adherent cells and supernatant were collected in one sample. Cells were counted, fixed, and washed as described before [58]. Cells were stained with 4 µM 4’,6-diamidino-2-fenylindool (DAPI) in PBS/1% Bovine serum albumin (BSA)/0.05% Tween20 for 30 min at room temperature and stored at 4 °C overnight. Readout and analysis were performed with the NucleoCounter NC-250 (Chemometec, Denmark), Winlist 3D Version 8, and Modfit Version 4.1.7 (Verity Software House, Topsham, ME, USA). Each analysis measured >10,000 single cell events with a coefficient of variation (CV)lower than 4. A statistical model with a polynomial S-phase shape showed the best fit (Reduced chi-square (RCS) between 1 and 3). Experiments were repeated three times.

### 4.6. Western Blotting

Chondrosarcoma cell lines were seeded in 6 well plates (9 × 10^4^ to 21 × 10^4^ cells/well) and allowed to attach overnight. Cultures were treated with talazoparib and H_2_O_2_ with concentrations ranging from 0 to 500 µM. At the indicated time points, cells were lysed in Hot SDS buffer (1% SDS, 10 mM Tris/Ethylenediaminetetraacetic acid (EDTA) pH 7.4) and supplemented with protease inhibitor cocktail and phosphatase inhibitor cocktail (Roche, Bazel, Switzerland). Urea (8 M) was added to the lysis buffer if samples were used to determine poly(ADP-ribosyl)ation (PARylation). Each sample (10 to 20 μg protein) was separated by SDS-PAGE and transferred to Polyvinylidene fluoride (PVDF) membranes using the Trans-Blot Turbo Transfer System (Bio-Rad Laboratories, Hercules, CA, USA). Blocking was performed with 5% non-fat dry milk (ELK) dissolved in tris-buffered saline with 0.1% Tween20 (TBS-T). Blots were examined for expression of cleaved caspase 3 (1:1000, clone 8G10, Cell Signaling Technology, Leiden, The Netherlands), cleaved PARP (1:1000, clone 46D11, Cell Signaling Technology), poly(ADP-ribose) (PAR) polymer (1:500, clone 10H, Abcam, Cambridge, UK), ataxia telangiectasia mutated (ATM) (1:500, clone D2E2, Cell Signaling Technology) and γ-H2AX (1:1000, clone 20E3, Cell Signaling Technology). The expression of α-tubulin (1:30000, clone DM1A, Sigma-Aldrich) was used as a loading control. Primary antibodies were diluted in TBS-T/5% BSA and incubated overnight at 4 °C. Blots were washed and incubated for 1 h at room temperature with horseradish peroxidase (HRP)-labelled-secondary antibodies diluted in TBS-T/5% ELK. Blots were developed with Pierce ECL Western Blotting Substrate (Thermo Fisher Scientific) and the ChemiDoc Touch Imaging System (Bio-Rad Laboratories). Images were analyzed and quantified with Image Lab Software Version 6 (Bio-Rad Laboratories).

### 4.7. RAD51 Foci Assay

CH2879, JJ012, and SW1353 cells were seeded on sterilized 3-Aminopropyltriethoxysilane (APES) coated slides (5 × 10^5^ to 1 × 10^6^ cells/slide). After overnight attachment, cultures were irradiated at 0 or 5 Gy of y-radiation using a ^137^C source (YXLON, Comet Technologies, Shelton, CT, USA), and were allowed to recover for 2 h or 24 h. Slides were fixed with 4% formaldehyde at 37 °C, washed with PBS/0.05% Tween20, and permeabilized with 100% ice-cold methanol at −20 °C as described previously [59]. Blocking was performed with PBS/1% BSA/0.05% Tween20 for 30 min at 37 °C. Slides were stained with primary antibodies against geminin (1:500, 10802-1-AP, Proteintech, Manchester, UK) and RAD51 (1:500, clone 14B4, GeneTex, Irvine, CA, USA) for 1 h at 37 °C. After washing, slides were incubated with an Alexa Fluor 488/549 secondary antibody mix supplemented with 0.5 µM Hoechst 33342 for 1 h at 37 °C. Slides were washed, covered with ProLong Gold Antifade Reagent (Invitrogen Life-Technologies), and a LSM 700 laser scanning confocal microscope (Carl Zeiss, Oberkochen, Germany) was used to acquire images of regions of interest. ZEN 2.3 Black software (Carl Zeiss) was used to export images. The number of RAD51 foci in each geminin-positive nucleus was automatically counted using a previously described ImageJ macro (IRIF analysis) [29]. Parameters were optimized for each cell line and results were validated by visual inspection of the images. At least 50 geminin-positive cells were counted per sample and a cell was considered RAD51 positive if >5 foci were detected in the nucleus [28].

### 4.8. Next Generation RNA Sequencing Analysis

RNA expression of *MGMT* in chondrosarcoma cell lines was determined from a next generation RNA sequencing dataset we previously generated [11]. The expression levels are shown in reads per kilobase per million (RPKM).

### 4.9. Methylation Array Analysis 

Methylation of *MGMT* in chondrosarcoma cell lines was determined from a previously described genome-wide methylation dataset (HumanMethylation450 BeadChip array, Illumina) [11]. The *MGMT* gene is located on chromosome 10 from base pair position 131,265,448 until 131,566,271 with a CpG island located in the promoter region ranging from 131,264,949 until 131,265,710 (USCS Genome Browser, GRCh37). Probes covering the gene and the promoter (i.e., 131,264,102 until 131,565,910) were selected to determine the methylation status of the samples.

### 4.10. Colony Formation Assay

CH2879, JJ012, and SW1353 cells were seeded in 10 cm dishes (4 × 10^2^ to 36 × 10^2^ cells/dish). After overnight attachment, cultures were treated with DMSO or 5 nM talazoparib and irradiated at 0, 1, 2, 4 or 6 Gy of y-radiation. Colonies were allowed to form for 1 to 2 weeks before dishes were washed with PBS and stained with 0.5% crystal violet/6% glutaraldehyde in H_2_O. An ImageJ macro was developed in-house to automatically quantify the number of colonies. Briefly, dishes were scanned and converted to 8-bit TIFF images to select the area of interest. Colonies were masked by thresholding of the images followed by a watershed segmentation to separate touching colonies. Colonies with a size ≤50 pixel^2^ were excluded from the analysis. Results were validated by visual inspection of the images. Surviving fractions were calculated by normalizing the data towards the plating efficiency (i.e., number of colonies/number of seeded cells) of the untreated control [60]. Experiments were performed in triplicate and repeated two times.

### 4.11. Statistical Calculations and Image Analysis

Significant changes between experimental groups were calculated with a Mann–Whitney test or a Kruskal–Wallis test followed by a Dunn’s post-hoc test. A Spearman correlation was performed to determine an association between two variables. Statistical analyses were performed in GraphPad Prism 8. The Bliss independence model (C = A + B – A × B) was used to predict synergy in treatment combinations, in which C represents the combined effect and A and B represent single agent effects [61,62]. Image analysis was performed with ImageJ V1.51 (National Institutes of Health, Bethesda, MD, USA). Heatmap figures were created with MORPHEUS (Broad Institute, Cambridge, MA, USA). 

## 5. Conclusions

In summary, inhibition of PARP combined with temozolomide or radiotherapy could be effective therapeutic strategies for both *IDH^WT^* and *IDH^MUT^* chondrosarcoma patients. Our study also establishes that a subset of chondrosarcomas may harbor a nonclassical DNA repair deficiency related to a low DNA damage sensing capacity, low *MGMT* expression, or a defect more downstream in the homologous recombination pathway. However, PARP inhibitor sensitivity could also be related to a change in the epigenetic landscape and further research is needed to elucidate the different mechanisms underlying PARP inhibitor sensitivity in chondrosarcoma. Taken together, our findings indicate that while PARP inhibitor sensitivity is multifactorial in chondrosarcoma, development of targeted therapies for chondrosarcoma remains an important endeavor, especially for the treatment of patients for which tumor resection is not a viable option. 

## Figures and Tables

**Table 1:** Growth corrected parameters (i.e., GR_50_ and GR_Inf_) and standard parameters (i.e., IC_50_ and E_Inf_) for talazoparib in chondrosarcoma cell lines.

Cell Line	Chondrosarcoma Subtype	*IDH* Mutation Status	GR_50_ (nM)	IC_50_ (nM)	GR_Inf_ (%)	E_Inf_ (%)
NDCS1	Dedifferentiated	Wildtype	34	25	−54	0
MCS170	Mesenchymal	Wildtype	75	-	7	57
SW1353	Central conventional	*IDH2* R172S	133	63	−25	3
HT1080	Dedifferentiated	*IDH1* R132C	188	61	10	11
CH3573	Central conventional	Wildtype	244	471	−2	26
L2975	Dedifferentiated	*IDH2* R172W	326	401	1	22
JJ012	Central conventional	*IDH1* R132G	371	193	−23	1
JJ012 + AGI-5198	Central conventional	“Wildtype”	659	303	−11	0
L3252B	Dedifferentiated	Wildtype	876	1442	−75	0
L835	Central conventional	*IDH1* R132C	1670	-	12	68
CH2879	Central conventional	Wildtype	1726	1103	−90	1
CH2879 + AGI-5198	Central conventional	Wildtype	4280	4060	−16	22

GR_50_ = the concentration of the drug at which growth rate inhibition (GR) = 0.5, equivalent of the IC_50_. GR_Inf_ = the effect of the drug at infinite concentration. GR_Inf_ lies between –1 and 1, equivalent of the E_Inf_ (maximum effect at infinite drug concentration).

**Figure A2:**
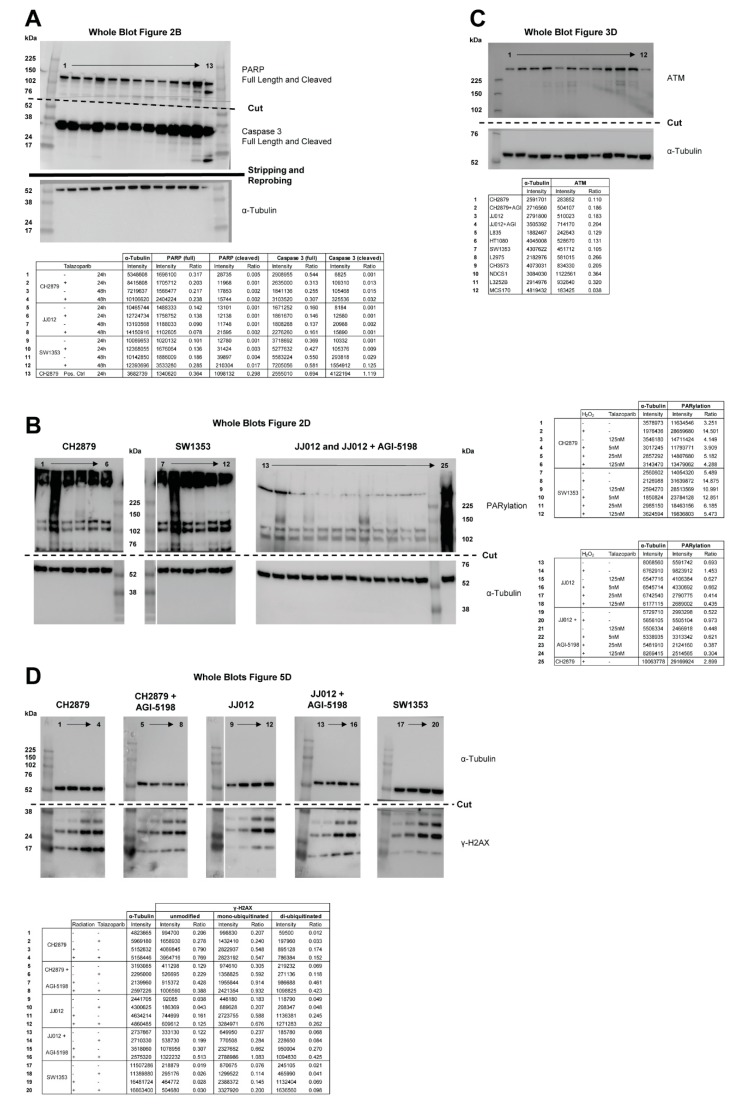
Whole blots with densitometry readings. (**A**) Whole blot with intensity values and calculated ratios of the Western blot depicted in Figure 2B. (**B**) Whole blots with intensity values and calculated ratios of the Western blots depicted in Figure 2D. (**C**) Whole blot with intensity values and calculated ratios of the Western blot depicted in Figure 3D. (**D**) Whole blots with intensity values and calculated ratios of the Western blots depicted in Figure 5D.

## References

[1] Hogendoorn P.C.W., Bovée J.V.M.G., Nielsen G.P., Fletcher C.D.M., Bridge J.A., Hogendoorn P.C.W., Mertens F. (2013). Chondrosarcoma (grades I-III), including primary and secondary variants and periosteal chondrosarcoma. WHO Classification of Tumours Soft Tissue and Bone.

[2] van Praag (Veroniek) V.M., Rueten-Budde A.J., Ho V., Dijkstra P.D.S., van der Geest I.C., Bramer J.A., Schaap G.R., Jutte P.C., Schreuder H.B., Ploegmakers J.J.W. (2018). Incidence, outcomes and prognostic factors during 25 years of treatment of chondrosarcomas. Surg. Oncol..

[3] Gelderblom H., Hogendoorn P.C.W., Dijkstra S.D., van Rijswijk C.S., Krol A.D., Taminiau A.H.M., Bovee J.V.M.G. (2008). The Clinical Approach Towards Chondrosarcoma. Oncologist.

[4] Amary M.F., Bacsi K., Maggiani F., Damato S., Halai D., Berisha F., Pollock R., O’Donnell P., Grigoriadis A., Diss T. (2011). IDH1 and IDH2 mutations are frequent events in central chondrosarcoma and central and periosteal chondromas but not in other mesenchymal tumours. J. Pathol..

[5] Pansuriya T.C., Van Eijk R., D’Adamo P., Van Ruler M.A.J.H., Kuijjer M.L., Oosting J., Cleton-Jansen A.M., Van Oosterwijk J.G., Verbeke S.L.J., Meijer D. (2011). Somatic mosaic IDH1 and IDH2 mutations are associated with enchondroma and spindle cell hemangioma in Ollier disease and Maffucci syndrome. Nat. Genet..

[6] Dang L., White D.W., Gross S., Bennett B.D., Bittinger M.A., Driggers E.M., Fantin V.R., Jang H.G., Jin S., Keenan M.C. (2009). Cancer-associated IDH1 mutations produce 2-hydroxyglutarate. Nature.

[7] Molenaar R.J., Radivoyevitch T., Maciejewski J.P., van Noorden C.J.F., Bleeker F.E. (2014). The driver and passenger effects of isocitrate dehydrogenase 1 and 2 mutations in oncogenesis and survival prolongation. Biochim. Biophys. Acta—Rev. Cancer.

[8] Xu W., Yang H., Liu Y., Yang Y., Wang P., Kim S.H., Ito S., Yang C., Wang P., Xiao M.T. (2011). Oncometabolite 2-hydroxyglutarate is a competitive inhibitor of α-ketoglutarate-dependent dioxygenases. Cancer Cell.

[9] Reitman Z.J., Jin G., Karoly E.D., Spasojevic I., Yang J., Kinzler K.W., He Y., Bigner D.D., Vogelstein B., Yan H. (2011). Profiling the effects of isocitrate dehydrogenase 1 and 2 mutations on the cellular metabolome. Proc. Natl. Acad. Sci. USA.

[10] Gagné L.M., Boulay K., Topisirovic I., Huot M.É., Mallette F.A. (2017). Oncogenic Activities of IDH1/2 Mutations: From Epigenetics to Cellular Signaling. Trends Cell Biol..

[11] Suijker J., Oosting J., Koornneef A., Struys E.A., Salomons G.S., Schaap F.G., Waaijer C.J.F., Wijers-Koster P.M., Briaire-de Bruijn I.H., Haazen L. (2015). Inhibition of mutant IDH1 decreases D-2-HG levels without affecting tumorigenic properties of chondrosarcoma cell lines. Oncotarget.

[12] Mardis E.R., Ding L., Dooling D.J., Larson D.E., McLellan M.D., Chen K., Koboldt D.C., Fulton R.S., Delehaunty K.D., McGrath S.D. (2009). Recurring Mutations Found by Sequencing an Acute Myeloid Leukemia Genome. N. Engl. J. Med..

[13] Yan H., Parsons D.W., Jin G., McLendon R., Rasheed B.A., Yuan W., Kos I., Batinic-Haberle I., Jones S., Riggins G.J. (2009). IDH1 and IDH2 Mutations in Gliomas. N. Engl. J. Med..

[14] Wang P., Wu J., Ma S., Zhang L., Yao J., Hoadley K.A., Wilkerson M.D., Perou C.M., Guan K.L., Ye D. (2015). Oncometabolite D-2-Hydroxyglutarate Inhibits ALKBH DNA Repair Enzymes and Sensitizes IDH Mutant Cells to Alkylating Agents. Cell Rep..

[15] Inoue S., Li W.Y., Tseng A., Beerman I., Elia A.J., Bendall S.C., Lemonnier F., Kron K.J., Cescon D.W., Hao Z. (2016). Mutant IDH1 Downregulates ATM and Alters DNA Repair and Sensitivity to DNA Damage Independent of TET2. Cancer Cell.

[16] Chan S.M., Thomas D., Corces-Zimmerman M.R., Xavy S., Rastogi S., Hong W.J., Zhao F., Medeiros B.C., Tyvoll D.A., Majeti R. (2015). Isocitrate dehydrogenase 1 and 2 mutations induce BCL-2 dependence in acute myeloid leukemia. Nat. Med..

[17] Karpel-Massler G., Ishida C.T., Bianchetti E., Zhang Y., Shu C., Tsujiuchi T., Banu M.A., Garcia F., Roth K.A., Bruce J.N. (2017). Induction of synthetic lethality in IDH1-mutated gliomas through inhibition of Bcl-xL. Nat. Commun..

[18] Tateishi K., Wakimoto H., Iafrate A.J., Tanaka S., Loebel F., Lelic N., Wiederschain D., Bedel O., Deng G., Zhang B. (2015). Extreme Vulnerability of IDH1 Mutant Cancers to NAD+ Depletion. Cancer Cell.

[19] Emadi A., Jun S.A., Tsukamoto T., Fathi A.T., Minden M.D., Dang C. (2014). V Inhibition of glutaminase selectively suppresses the growth of primary acute myeloid leukemia cells with IDH mutations. Exp. Hematol..

[20] Sulkowski P.L., Corso C.D., Robinson N.D., Scanlon S.E., Purshouse K.R., Bai H., Liu Y., Sundaram R.K., Hegan D.C., Fons N.R. (2017). 2-Hydroxyglutarate produced by neomorphic IDH mutations suppresses homologous recombination and induces PARP inhibitor sensitivity. Sci. Transl. Med..

[21] Molenaar R.J., Radivoyevitch T., Nagata Y., Khurshed M., Przychodzen B., Makishima H., Xu M., Bleeker F.E., Wilmink J.W., Carraway H.E. (2018). Idh1/2 mutations sensitize acute myeloid leukemia to parp inhibition and this is reversed by idh1/2-mutant inhibitors. Clin. Cancer Res..

[22] Turcan S., Fabius A.W., Borodovsky A., Pedraza A., Brennan C., Huse J., Viale A., Riggins G.J., Chan T.A. (2013). Efficient induction of differentiation and growth inhibition in IDH1 mutant glioma cells by the DNMT Inhibitor Decitabine. Oncotarget.

[23] Borodovsky A., Salmasi V., Turcan S., Fabius A.W.M., Baia G., Eberhart C.G., Weingart J.D., Gallia G.L., Baylin S.B., Chan T.A. (2013). 5-azacytidine reduces methylation, promotes differentiation and induces tumor regression in a patient-derived IDH1 mutant glioma xenograft. Oncotarget.

[24] Shen Y., Aoyagi-Scharber M., Wang B. (2015). Trapping Poly(ADP-Ribose) Polymerase. J. Pharmacol. Exp. Ther..

[25] Shen Y., Rehman F.L., Feng Y., Boshuizen J., Bajrami I., Elliott R., Wang B., Lord C.J., Post L.E., Ashworth A. (2013). BMN673, a novel and highly potent PARP1/2 inhibitor for the treatment of human cancers with DNA repair deficiency. Clin. Cancer Res..

[26] Engert F., Kovac M., Baumhoer D., Nathrath M., Fulda S. (2017). Osteosarcoma cells with genetic signatures of BRCAness are susceptible to the PARP inhibitor talazoparib alone or in combination with chemotherapeutics. Oncotarget.

[27] Wilkerson P.M., Dedes K.J., Samartzis E.P., Dedes I., Lambros M.B., Natrajan R., Gauthier A., Piscuoglio S., Töpfer C., Vukovic V. (2017). Preclinical evaluation of the PARP inhibitor BMN-673 for the treatment of ovarian clear cell cancer. Oncotarget.

[28] Naipal K.A.T., Verkaik N.S., Ameziane N., Van Deurzen C.H.M., Ter Brugge P., Meijers M., Sieuwerts A.M., Martens J.W., O’Connor M.J., Vrieling H. (2014). Functional Ex vivo assay to select homologous recombination-deficient breast tumors for PARP inhibitor treatment. Clin. Cancer Res..

[29] Typas D., Luijsterburg M.S., Wiegant W.W., Diakatou M., Helfricht A., Thijssen P.E., Van De Broek B., Mullenders L.H., Van Attikum H. (2015). The de-ubiquitylating enzymes USP26 and USP37 regulate homologous recombination by counteracting RAP80. Nucleic Acids Res..

[30] Gill S.J., Travers J., Pshenichnaya I., Kogera F.A., Barthorpe S., Mironenko T., Richardson L., Benes C.H., Stratton M.R., McDermott U. (2015). Combinations of PARP inhibitors with temozolomide drive PARP1 trapping and apoptosis in Ewing’s sarcoma. PLoS ONE.

[31] Smith M.A., Reynolds C.P., Kang M.H., Kolb E.A., Gorlick R., Carol H., Lock R.B., Keir S.T., Maris J.M., Billups C.A. (2015). Synergistic activity of PARP inhibition by talazoparib (BMN 673) with temozolomide in pediatric Cancer Models in the Pediatric Preclinical Testing Program. Clin. Cancer Res..

[32] Van Nifterik K.A., van den Berg J., van der Meide W.F., Ameziane N., Wedekind L.E., Steenbergen R.D.M., Leenstra S., Lafleur M.V.M., Slotman B.J., Stalpers L.J.A. (2010). Absence of the *MGMT* protein as well as methylation of the *MGMT* promoter predict the sensitivity for temozolomide. Br. J. Cancer.

[33] Ramakrishnan V., Kushwaha D., Koay D.C., Reddy H., Mao Y., Zhou L., Ng K., Zinn P., Carter B., Chen C.C. (2011). Post-transcriptional regulation of O 6 -methylguanine-DNA methyltransferase *MGMT* in glioblastomas. Cancer Biomark..

[34] Fertil B., Malaise E.P. (1981). Inherent cellular radiosensitivity as a basic concept for human tumor radiotherapy. Int. J. Radiat. Oncol. Biol. Phys..

[35] Lu Y., Kwintkiewicz J., Liu Y., Tech K., Frady L.N., Su Y.T., Bautista W., Moon S.I., MacDonald J., Ewend M.G. (2017). Chemosensitivity of IDH1-mutated gliomas due to an impairment in PARP1-mediated DNA repair. Cancer Res..

[36] Tateishi K., Higuchi F., Miller J.J., Koerner M.V.A., Lelic N., Shankar G.M., Tanaka S., Fisher D.E., Batchelor T.T., Iafrate A.J. (2017). The alkylating chemotherapeutic temozolomide induces metabolic stress in IDH1-mutant cancers and potentiates NAD+depletion-mediated cytotoxicity. Cancer Res..

[37] De Jong Y., Monderer D., Brandinelli E., Monchanin M., van den Akker B.E., van Oosterwijk J.G., Blay J.Y., Dutour A., Bovée J.V.M.G. (2018). Bcl-xl as the most promising Bcl-2 family member in targeted treatment of chondrosarcoma. Oncogenesis.

[38] Peterse E.F.P., van den Akker B.E.W.M., Niessen B., Oosting J., Suijker J., de Jong Y., Danen E.H.J., Cleton-Jansen A.-M., Bovée J.V.M.G. (2017). NAD Synthesis Pathway Interference Is a Viable Therapeutic Strategy for Chondrosarcoma. Mol. Cancer Res..

[39] Peterse E.F.P., Niessen B., Addie R.D., De Jong Y., Cleven A.H.G., Kruisselbrink A.B., Van Den Akker B.E.W.M., Molenaar R.J., Cleton-Jansen A.M., Bovée J.V.M.G. (2018). Targeting glutaminolysis in chondrosarcoma in context of the IDH1/2 mutation. Br. J. Cancer.

[40] Núñez F.J., Mendez F.M., Kadiyala P., Alghamri M.S., Savelieff M.G., Garcia-Fabiani M.B., Haase S., Koschmann C., Calinescu A.-A., Kamran N. (2019). IDH1-R132H acts as a tumor suppressor in glioma via epigenetic up-regulation of the DNA damage response. Sci. Transl. Med..

[41] Hafner M., Niepel M., Chung M., Sorger P.K. (2016). Growth rate inhibition metrics correct for confounders in measuring sensitivity to cancer drugs. Nat. Methods.

[42] Smeenk G., Wiegant W.W., Marteijn J.A., Luijsterburg M.S., Sroczynski N., Costelloe T., Romeijn R.J., Pastink A., Mailand N., Vermeulen W. (2012). Poly(ADP-ribosyl)ation links the chromatin remodeler SMARCA5/SNF2H to RNF168-dependent DNA damage signaling. J. Cell Sci..

[43] Ciccarone F., Zampieri M., Caiafa P. (2017). PARP1 orchestrates epigenetic events setting up chromatin domains. Semin. Cell Dev. Biol..

[44] Newton M.D., Makovets S., Krejci L., Kotenko O., Altmannova V., Vasianovich Y. (2016). Unloading of homologous recombination factors is required for restoring double-stranded DNA at damage repair loci. EMBO J..

[45] de Jong Y., Ingola M., Briaire-de Bruijn I.H., Kruisselbrink A.B., Venneker S., Palubeckaite I., Heijs B.P.A.M., Cleton-Jansen A.-M., Haas R.L.M., Bovée J.V.M.G. (2019). Radiotherapy resistance in chondrosarcoma cells; a possible correlation with alterations in cell cycle related genes. Clin. Sarcoma Res..

[46] Wei H., Yu X. (2016). Functions of PARylation in DNA Damage Repair Pathways. Genom. Proteom. Bioinfo..

[47] Césaire M., Ghosh U., Austry J.-B., Muller E., Cammarata F.P., Guillamin M., Caruso M., Castéra L., Petringa G., Cirrone G.A.P. (2019). Sensitization of chondrosarcoma cells with PARP inhibitor and high-LET radiation. J. Bone Oncol..

[48] Murai J., Zhang Y., Morris J., Ji J., Takeda S., Doroshow J.H., Pommier Y. (2014). Rationale for Poly(ADP-ribose) Polymerase (PARP) Inhibitors in Combination Therapy with Camptothecins or Temozolomide Based on PARP Trapping versus Catalytic Inhibition. J. Pharmacol. Exp. Ther..

[49] de Bono J., Ramanathan R.K., Mina L., Chugh R., Glaspy J., Rafii S., Kaye S., Sachdev J., Heymach J., Smith D.C. (2017). Phase I, dose-escalation, two-part trial of the PARP inhibitor talazoparib in patients with advanced germline BRCA1/2 mutations and selected sporadic cancers. Cancer Discov..

[50] Brada M., Judson I., Beale P., Moore S., Reidenberg P., Statkevich P., Dugan M., Batra V., Cutler D. (1999). Phase I dose-escalation and pharmacokinetic study of temozolomide (SCH 52365) for refractory or relapsing malignancies. Br. J. Cancer.

[51] Gil-Benso R., Lopez-Gines C., López-Guerrero J.A., Carda C., Callaghan R.C., Navarro S., Ferrer J., Pellín A., Llombart-Bosch A. (2003). Establishment and characterization of a continuous human chondrosarcoma cell line, ch-2879: Comparative histologic and genetic studies with its tumor of origin. Lab. Investig..

[52] Scully S.P., Berend K.R., Toth A., Qi W.N., Qi Z., Block J.A. (2000). Interstitial collagenase gene expression correlates with in vitro invasion in human chondrosarcoma. Clin. Orthop. Relat. Res..

[53] Calabuig-Fariñas S., Benso R.G., Szuhai K., Machado I., López-Guerrero J.A., De Jong D., Peydró A., Miguel T.S., Navarro L., Pellín A. (2012). Characterization of a new human cell line (CH-3573) derived from a grade II chondrosarcoma with matrix production. Pathol. Oncol. Res..

[54] van Oosterwijk J.G., de Jong D., van Ruler M.A., Hogendoorn P.C., Dijkstra P.D.S., van Rijswijk C.S., Machado I., Llombart-Bosch A., Szuhai K., Bovée J.V. (2012). Three new chondrosarcoma cell lines: One grade III conventional central chondrosarcoma and two dedifferentiated chondrosarcomas of bone. BMC Cancer.

[55] Kudo N., Ogose A., Hotta T., Kawashima H., Gu W., Umezu H., Toyama T., Endo N. (2007). Establishment of novel human dedifferentiated chondrosarcoma cell line with osteoblastic differentiation. Virchows Arch..

[56] Rasheed S., Nelson-Rees W.A., Toth E.M., Arnstein P., Gardner M.B. (1974). Characterization of a newly derived human sarcoma cell line (HT-1080). Cancer.

[57] De Jong Y., Van Maldegem A.M., Marino-Enriquez A., De Jong D., Suijker J., Briaire-De Bruijn I.H., Kruisselbrink A.B., Cleton-Jansen A.M., Szuhai K., Gelderblom H. (2016). Inhibition of Bcl-2 family members sensitizes mesenchymal chondrosarcoma to conventional chemotherapy: Report on a novel mesenchymal chondrosarcoma cell line. Lab. Investig..

[58] Van Haaften C., Boot A., Corver W.E., Van Eendenburg J.D., Trimbos B.J., Van Wezel T. (2015). Synergistic effects of the sesquiterpene lactone, EPD, with cisplatin and paclitaxel in ovarian cancer cells. J. Exp. Clin. Cancer Res..

[59] Corver W.E., Demmers J., Oosting J., Sahraeian S., Boot A., Ruano D., Van Wezel T., Morreau H. (2018). ROS-induced near-homozygous genomes in thyroid cancer. Endocr. Relat. Cancer.

[60] Franken N.A.P., Rodermond H.M., Stap J., Haveman J., van Bree C. (2006). Clonogenic assay of cells in vitro. Nat. Protoc..

[61] Greco W., Bravo G., Parsons J. (1995). The search for Synergy: A critical review from a repsonse persepective. Pharmacol. Rev..

[62] Borisy A.A., Elliott P.J., Hurst N.W., Lee M.S., Lehar J., Price E.R., Serbedzija G., Zimmermann G.R., Foley M.A., Stockwell B.R. (2003). Systematic discovery of multicomponent therapeutics. Proc. Natl. Acad. Sci. USA.

